# Developing A Conceptual Framework for Early Intervention Vocational Rehabilitation for People Following Spinal Cord Injury

**DOI:** 10.1007/s10926-022-10060-9

**Published:** 2022-08-04

**Authors:** Jennifer A. Dunn, R. A. Martin, J. J. Hackney, J. L. Nunnerley, D. L. Snell, J. A. Bourke, T. Young, A. Hall, S. Derrett

**Affiliations:** 1 Department of Orthopaedic Surgery and Musculoskeletal Medicine, University of Otago, Christchurch, New Zealand; 2 Burwood Academy Trust, Burwood Hospital, Christchurch, New Zealand; 3 Rehabilitation Teaching and Research Unit, Department of Medicine, University of Otago, Wellington, New Zealand; 4 Menzies Health Institute, Griffith University, Queensland, Australia; 5 New Zealand Spinal Trust, Burwood Hospital, Christchurch, New Zealand; 6 Ngāi Tahu Māori Health Research Unit, Preventive and Social Medicine, University of Otago, Dunedin, New Zealand

**Keywords:** Vocational rehabilitation, Spinal cord injury, Return to work

## Abstract

**Purpose:**

Early intervention vocational rehabilitation (EIVR) can improve return to work (RTW) outcomes for people with spinal cord injury (SCI). However, mechanisms explaining how and why EIVR works are not well understood. This study aims to develop a conceptual framework describing key mechanisms of EIVR intervention effect following SCI.

**Methods:**

We synthesised data from a realist literature review with data from interviews of people with SCI (n = 30), a survey of people with SCI who had received EIVR (n = 37), a focus group of EIVR providers and a focus group of community vocational providers. We first synthesised the literature review and interviews to develop an initial programme theory describing the contexts in which mechanisms are activated to produce EIVR outcomes. Then we used data from the survey and focus groups to further refine the EIVR programme theory. Finally, a conceptual framework was developed to support knowledge dissemination.

**Results:**

By ensuring consistent messaging across the multi-disciplinary team, EIVR programmes establish and maintain hope that work is possible following injury. Conversations about work allow individuals to determine the priority of work following injury. These conversations can also improve self-efficacy by providing individualized support to envisage pathways toward RTW goals and maintain worker identity. The synthesised study findings highlight the contexts and resources required to trigger activation of these mechanisms.

**Conclusions:**

EIVR key mechanisms of effect are not specific to SCI as a health condition, therefore enabling this framework to be applied to other populations who face similar impairments and return to work barriers.

## Introduction

Vocational rehabilitation following injury, when available, is usually provided once an individual has completed inpatient and/or outpatient rehabilitation and is ready to look at returning to work.[1] However, returning to work after lengthy rehabilitation is difficult for many people who sustain a serious injury such as spinal cord injury (SCI). Return to work (RTW) rates are low for people with SCI, with approximately 30% of people returning to work after SCI and RTW on average five years following injury.[2, 3] Early intervention vocational rehabilitation (EIVR) was developed in response to such low RTW rates and has been in practice in spinal rehabilitation units in New Zealand (NZ) and Australia for a number of years with promising results.[4–6] EIVR aims to improve long term employment outcomes for people with SCI by early fostering of hope, optimising self-efficacy and maintaining worker identity.[7] It is based on the knowledge that some people, even after serious injury such as SCI, start making decisions about work within a month of injury.[8] Krause et al.[3] found the length of time from SCI to RTW can be shortened by up to five years when individuals return to their pre-injury employer; other studies have supported this finding highlighting the specific value of returning to a pre-injury employer.[5, 9] EIVR includes liaison with current employers following injury, providing information and support, and reinforcing early and ongoing communication between employer and employee.[5] In contrast, other studies have found that some individuals were not ready to consider RTW for reasons such as prioritising their physical rehabilitation above RTW and those unable to conceive RTW as a possibility in their future.[9] Thus, there is a need to understand how different people respond to the resources provided within EIVR to ensure that vocational interventions are individualised, timely and sensitive to the person receiving the intervention.

A conceptual framework explains, either graphically or in narrative form, the key factors or constructs and the presumed relationships between them.[10] Conceptual frameworks are developed to describe phenomena that occur under similar conditions.[11] This study aimed to develop a conceptual framework describing the mechanisms of EIVR within the SCI population. This will facilitate better understanding of how EIVR works with people with SCI, and what individual and rehabilitation team contexts might be required to best support the achievement of RTW outcomes in this population.

## Methods

This study is a synthesis of information sitting within a broader study called the Early Vocational rehabilitation in neurological conditions Study (EVocS), a realist mixed-methods study examining the contexts and mechanisms of effect influencing RTW outcomes among people with neurological impairments. Ethical approval for the EVocS study was gained from the University of Otago Ethics Committee (Health) (H19/170).

**Fig. 1:**
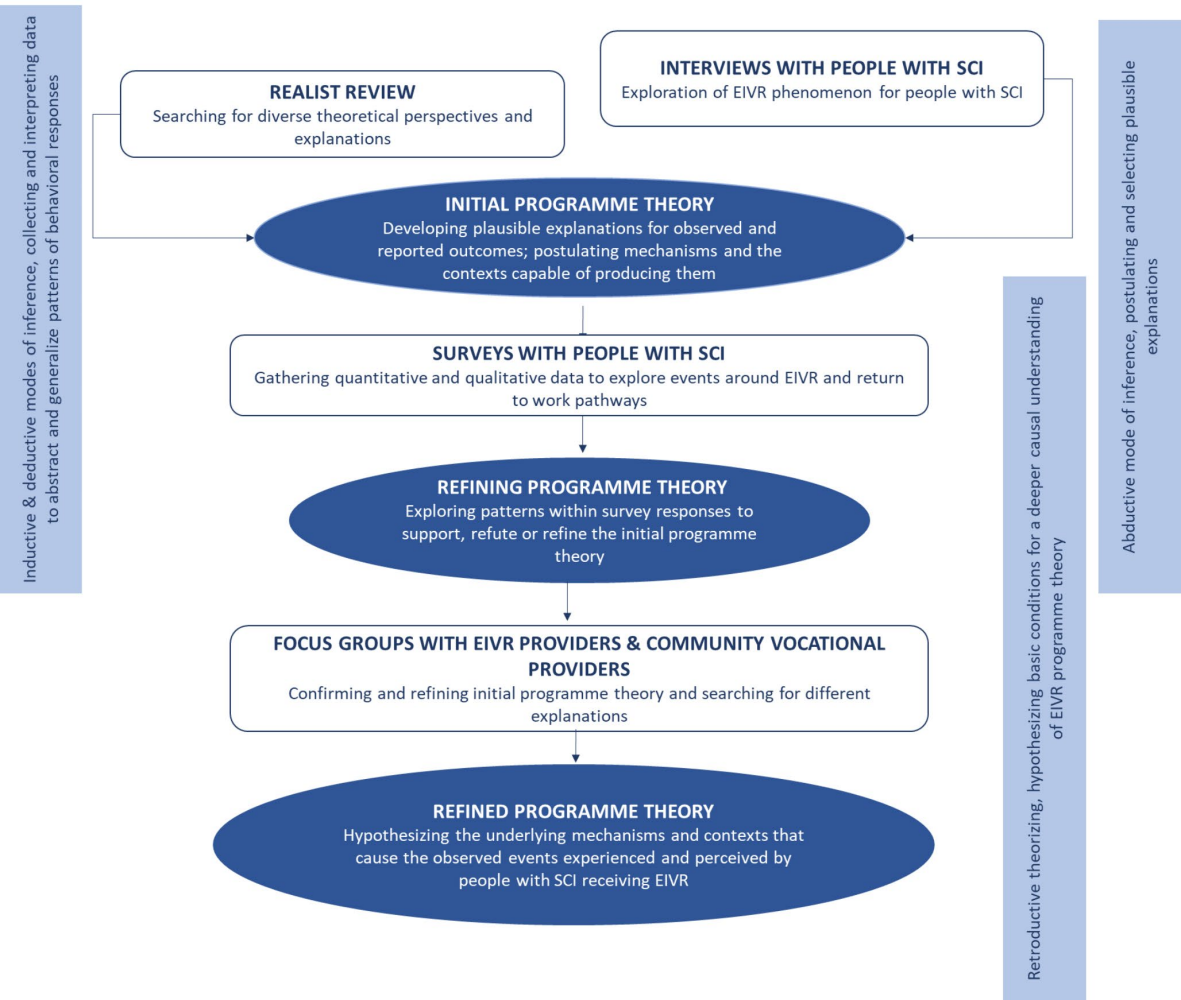
Steps and modes of inference of synthesis process

To develop the EIVR programme theory and resulting conceptual framework we synthesised findings from a realist review of the literature and interviews of people with SCI (Fig. 1). Then data from a survey of people with SCI, two focus groups (one with vocational providers for people with SCI (not EIVR) and one with EIVR providers from the New Zealand Spinal Trust) as well as interviews of vocational providers who were unable to attend the focus groups (n = 2) were used to refine the framework. The protocol for this study [12], as well as methods and results of the realist review of the literature [7], interview study [13] and survey [14] have been previously published. Key steps however are outlined in brief below.

Realist review of the literature: Using a rough initial programme theory developed with EIVR providers as a guide, we conducted the following steps: literature search, article selection, extracting and data organising, synthesis of evidence and programme theory refinement. In February 2020, we conducted searches of the literature, including grey literature of reports and policy documents from key international organisational websites. We searched for published literature using the following databases: Cinahl, Embase, EMcare, Medline, PsychInfo and Scopus. Google Scholar was used to search for grey literature. Extracted data were inductively categorised into conceptual categories and subsequently, potential contexts (C), mechanisms (M) and outcomes (O) using a realist explanatory logic.[15] By iteratively analysing whether the Context-Mechanisms-Outcome configurations (CMOC’s) generated from the literature review supported, refuted or refined the rough programme theory we sought to understand each CMO’s place and relationship within the programme theory.[7].

Interviews of people with SCI: Between April and July 2020 we conducted one-on-one interviews with 30 people with SCI who had received EIVR via NZ Spinal Trust Vocational Rehabilitation Services. The aim of the interview study was to develop a theoretical understanding of how people with SCI respond to EIVR and the contexts in which mechanisms of intervention effect were likely to be achieved. Interviews were coded independently by three team members (RM, JN and TY) following a priori determined code headings: (a) intervention resources, actions and tasks, (b) contextual factors, (c) mechanisms, and (d) outcomes. Particular attention was paid to data extracts that provided evidence of links between the code headings. We used manual diagramming to bring together, summarise and refine key CMO configurations of the developing theory about how, and in what contexts, EIVR works for a range of people following SCI.[13].

Survey of people with SCI: We conducted a cross-sectional survey of 37 people with SCI recruited from NZ Spinal Trust Vocational Rehabilitation Service who had sustained an SCI within the previous five years. The aims of the survey were to describe demographic and clinical characteristics and RTW outcomes of people with SCI who had received EIVR and identify key EIVR mechanisms. Data were analysed using IBM SPSS Statistics for Windows, Version 26.0. Contingency tables (cross-tabs) were calculated for discrete variables and chi-squared tests determined significance of 2-way associations. For continuous variables, independent-samples t-tests were used. Results are descriptive and reported as frequencies, percentages, means and standard deviations, medians and interquartile ranges, and odds ratios (OR) with 95% confidence intervals (CI), as appropriate. The free text responses at the end of the survey were analysed using qualitative description.[16] Ideas emerging from the written responses were categorised by one researcher (DS) and reviewed by the team. First, responses were collated, read and re-read and then grouped into preliminary categories and then grouped together based on similarities to form a smaller number of categories. These were then revised and refined again following discussion between the research team. The quantitative survey results and qualitative free text responses were integrated using a triangulation approach.[17] After component data were separately analysed we looked for agreement, disagreement or silence across data from the respective methods. We particularly looked at integrating quantitative findings related to mechanisms of EIVR with free text responses because this was most relevant to our research questions.

Focus groups with EIVR providers: To further confirm and refine the development of the initial programme theory and to check for ongoing relevance, in November 2020 we conducted a focus group with five NZ Spinal Trust staff currently providing EIVR to people with SCI.

Focus groups with vocational providers: In December 2020, we conducted one focus group with three participants and two individual interviews with community-based vocational providers who had experience working with people following SCI. This data further contributed to our understanding of the broader context in which EIVR services are currently situated, and how people with SCI access vocational support after EIVR.

Synthesis: To integrate the findings from the different components of the study we used a process similar to that described by Salter and Kothari.[18] Fig. 1 overviews the various steps and modes of inference applied at various stages across the research to postulate, explore, test and select the most plausible generative mechanisms explaining how contexts are capable of producing outcomes experienced by people with SCI receiving EIVR. First, using data gathered and analysed from the literature review and interviews, an initial programme theory was developed postulating mechanisms and the contexts capable of activating them. In this step JH, JD and RM triangulated data from the literature review and interviews with people following SCI, developing a matrix that included CMOC’s from Phases 1 and 2, key themes and supporting evidence (Online resource 1). Second, data from surveys was used to explore events around EIVR and return to work pathways, with additional analytic findings and evidence being added to the matrix. Finally, data gathered from EIVR providers and community-based vocational providers were used to further test and refine the programme theory. Using the matrix to guide discussions, JH, JD and RM met to articulate and then visualise a metaphor depicting the key mechanisms of effect within the refined programme theory. Following this, meetings with all team members were used to present and refine the conceptual framework. Lastly JH, JD and RM worked with a visual designer to develop the figure illustrating the conceptual framework.

## Results

The mean age (SD, range) of the interview study sample was 52 years (11 years, 21–67 years) and the survey study sample was 50 years (15 years, 18–71 years). The demographic and clinical characteristics of the interview and survey participants are presented in Table 1. The most noticeable difference between the interview and survey participants was the level of SCI, with the majority of survey participants being paraplegic (63%) compared to 40% in the interview study. As shown in Table 1, the gender mix for the interview participants (67% male) and the survey participants (49% male) were lower than that reported for the NZ SCI population (72% male) [19]. Fewer participants had returned to work following their SCI in the interview study (43%) than the survey study (54%). Of those who had returned to work in the survey study, just over half (58%) reported they returned to work within 12 months of their SCI, and most had remained in the same role with the same employer (74%). Considering associations between RTW outcomes and demographic and clinical variables, although the numbers are small, those in management roles had higher odds of returning to work within 12 months of injury (OR = 3.8, 95% CI 0.6, 22.3; p = 0.04), to the same employer (OR = 4.2, 95% CI 0.7, 24.5; p = 0.01) and to the same role (OR = 3.3, 95% CI 0.6, 20.17; p = 0.09) compared with those in non-management roles. There were no associations identified between RTW outcomes and demographic or clinical variables such as age, gender, ethnicity, educations, SCI type, rehabilitation funder or mobility status.

**Table 1:** Demographic and clinical characteristics of the study population with reference to the wider NZ SCI population

	Survey study (n = 37) n (%)^1^	Interview study (n = 30) n (%)	NZ SCI Population^2^ (%)
**Demographic characteristics**			
Sex (male)	18 (49)	20 (67)	72%
Ethnicity New Zealand European New Zealand Māori Other	28 (76) 7 (19) 2 (5)	18 (60) 3 (10) 9 (30)	48% 22% 30%
**Clinical Characteristics**			
SCI cause (traumatic)	28 (76)	26 (87)	68%
SCI type Tetraplegia Paraplegia	11 (30) 23 (63)	18 (60) 12 (40)	
Mobility status Full-time wheelchair user Mix of wheelchair and walking Community walker	17 (46) 2 (5) 18 (49)	12 (40) 5 (17) 13 (43)	
Returned to work following SCI (yes)	19 (54)	13 (43)	

(1) Variables, where percentages reported do not sum to 100, reflect missing data; (2) New Zealand Spinal Cord Injury Registry 2020; SCI = spinal cord impairment/injury

To explore EIVR resources provided to the survey participants by NZ Spinal Trust Vocational Service, we analysed survey data by looking at participants who had and had not returned to work following SCI. Findings showed hopefulness about returning to work differentiated the two groups, with those who had returned to work indicating that they felt hopeful about RTW while they were still in the spinal rehabilitation unit (84%). Although the numbers are small, participants who had returned to work were also more likely to have endorsed feeling positive/optimistic about RTW (79%) and as having positive expectations about RTW (74%) compared to those who did not RTW (33%) respectively. There was an association between feeling hopeful about returning to work while still in the spinal unit and RTW outcome (OR = 2.25, 95% CI 1.13, 4.50; p = 0.04). There were non-significant trends to those finding support to talk through options, discuss realistic expectations, explore adaptations, and successfully returning to work. These findings and EIVR resources survey participants endorsed as accessing are summarised in Table 2.

**Table 2:** Return to work outcomes and EIVR resources accessed by participants (n = 37)

	Whole Sample (n = 37) N (%)	RTW (n = 19) N (%)	No RTW (n = 15) N (%)
I was approached by the NZST team at the right time to discuss return to work (n (%), yes)^1^	19 (51.4)	12 (63.2)	7 (46.6)
I felt hopeful about returning to work while I was still in the spinal unit (n (%), yes)	24 (64.9)	16 (84.2)	8 (53.3)
I expected to return to work after my SCI (n (%), yes)	24 (64.9)	12 (63.2)	9 (60.0)
Specific NZST resources (n, yes):			
- NZST helped me feel positive/optimistic about returning to work	23 (62.2)	15 ( (78.9)	8 (53.3)
- NZST helped me work through future return to work options	22 (59.5)	12 (63.2)	10 (66.7)
- NZST talked about how returning to work might work/what to expect	19 (51.4)	14 (73.7)	5 (33.3)
**-** NZST helped me look at adaptations for returning to work	15 (40.5)	11 (57.9)	4 (26.7)
- NZST helped me think about returning to work by sharing others’ stories	14 (37.8)	8 (42.1)	6 (40.0)
- NZST helped me sort through and come up with options	14 (37.8)	9 (47.4)	5 (33.3)
- NZST helped me talk about return to work goals with the multidisciplinary team	14 (37.8)	8 (42.1)	6 (40.0)
- NZST gave me information about future work or study options	13 (35.1)	7 (36.8)	6 (40.0)
- NZST helped me to understand administration processes for return to work	12 (32.4)	6 (31.6)	6 (40.0)
- NZST talked to family/Whānau re my return to work possibilities	9 (24.3)	5 (26.3)	4 (26.7)
- NZST coordinated return to work administration for me	8 (21.6)	5 (26.3)	3 (20.0)
- NZST talked to my employer on my behalf	7 (18.9)	4 (21.1)	3 (20.0)
- NZST offered practical support to apply for jobs	5 13.5)	2 (10.5)	3 (20.0)
- NZST helped me talk to my employer	0 (0.0)	0 (0.0)	0 (0.0)

1. Yes = agree or strongly agree; NZST = New Zealand Spinal Trust Vocational Rehabilitation Service (EIVR provider); RTW = return to work; SCI = spinal cord injury/impairment

### Conceptual framework

To develop the conceptual framework we articulated the results of our analysis using a gardening metaphor, encapsulating the varying types and degrees of growth and support an individual needed in order to think about, plan and action RTW following SCI.

**Fig. 2:**
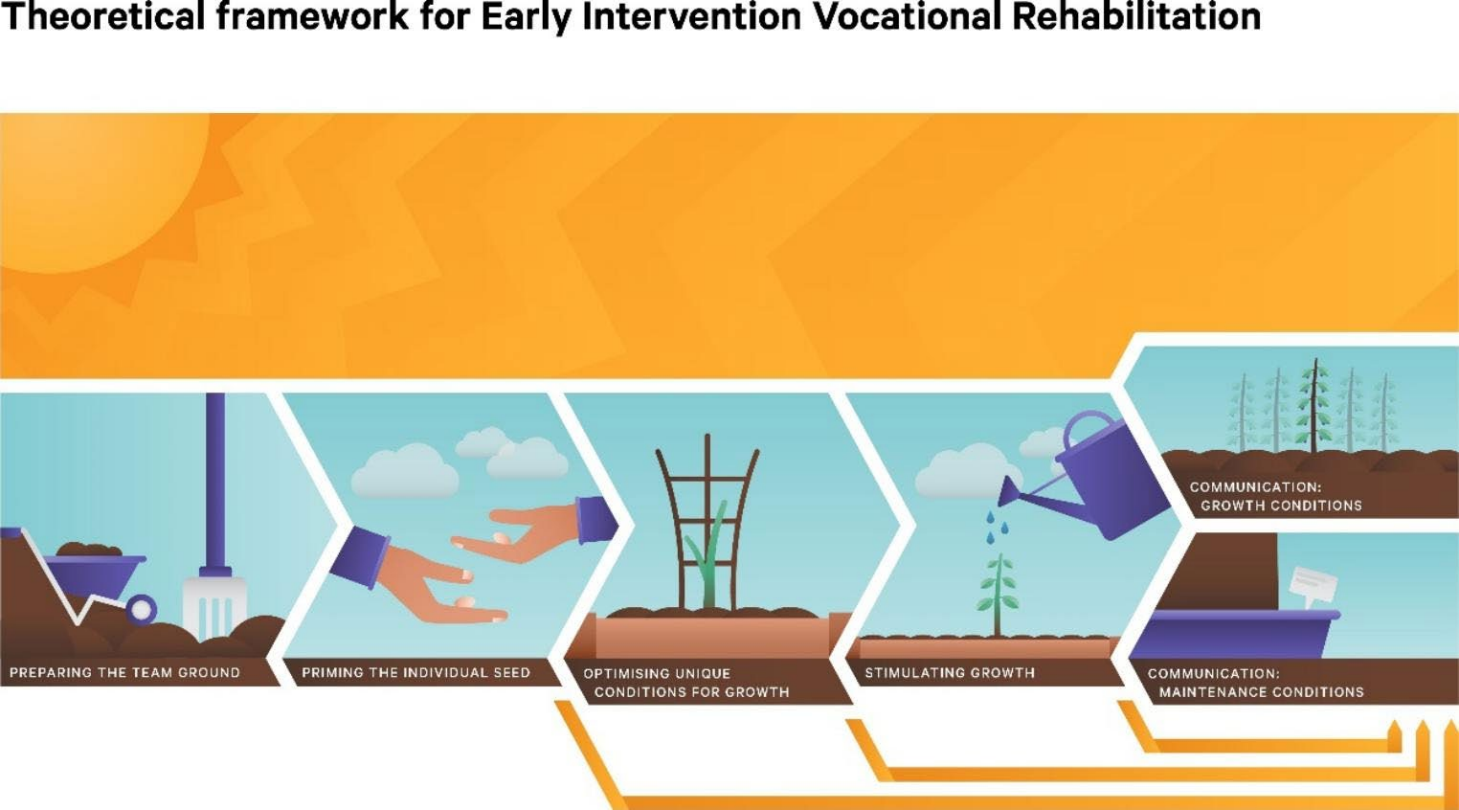
Conceptual framework describing Early Intervention Vocational Rehabilitation

We conceptualised the individual receiving EIVR as a seed. This metaphor has been used in previous literature describing EIVR.[20] A seed has potential for growth, given appropriate and continual conditions that can be altered depending on need. Figure 2 summarises the conceptual framework describing EIVR. There are seven key concepts: hope, preparing the team ground, priming the individual seed, optimising unique conditions for growth, stimulating growth, communication – growth conditions and communication – maintenance conditions. These concepts are described in more detail below.

### Hope

Results from analyses across all data sources identified the cornerstone of an EIVR programme is hope. Findings identified that the potential to work is within the individual, but that it requires energy to commence the process and sustain change. Linking to the gardening metaphor, for a seed to grow it requires sunlight, water and gases. These three elements are converted to energy to enable growth in a process called photosynthesis. We postulate that hope is to EIVR as sunlight is to photosynthesis. If the other conditions are present, then hope becomes the process by which elements of the EIVR process are converted into goals and pathways to facilitate RTW following injury. This energy/hope can be used immediately or can be stored for use later, as the need arises. This is particularly important as an individual will require different levels of energy/input depending on stages and setbacks throughout the rehabilitation process. We have attempted to demonstrate hope within the model with graphical representation of the sun being synonymous with hope encompassing the entire EIVR process. The inclusion of clouds in places illustrates that at times hopefulness about RTW can fluctuate.

### Preparing the team ground

It is important that the rehabilitation environment is open for RTW conversations to commence during early stages of rehabilitation. If RTW goals and consideration of the RTW process are discussed and established during the initial rehabilitation stages through positive expectations of RTW from all members of the rehabilitation team, then hope and positive perceptions about RTW are reinforced for the individual. Consistent team messaging throughout the rehabilitation continuum about RTW being a possibility at some time is important for the establishment, maintenance and optimising of hope.

### Priming the individual seed

The individualised nature of EIVR enables optimal person-specific conditions for each individual to be established. This allows the individual to consider their own RTW goals. Much of this process is based on development of the relationship between the EIVR provider and the individual, specifically that the EIVR provider is someone the individual trusts. The potential value of lived experience of the EIVR provider was also underscored. EIVR acknowledges that different levels of input are required for each individual, with the EIVR provider ensuring that the rehabilitation environment is optimised to support differences in readiness and adjustment. This is achieved by exploring with the individual who they are, establishing their biographical continuity following onset of their illness/injury and their expectations of work within the wider cultural context. Accepting the individual can stay in this or any subsequent stage for as long as required is important. People with SCI may remain in this space throughout their rehabilitation stay. EIVR providers would then work with the individual to enable transition to further stages of EIVR once they are ready, or if the individual is not ready at the time of inpatient discharge, communicate with community-based vocational consultants to ensure continuation of services. This latter process is illustrated in the model by the yellow arrows that go from any of the stages to the ‘communication maintenance conditions’ stage.

### Optimising unique conditions for growth

If the environmental conditions are right and the individual is ready to engage in EIVR, the EIVR provider, through their established relationship, identifies the type of support the individual requires to promote self-efficacy and enable retention of worker identity through an individualised and strengths-based approach. Internal schema and psychological coping factors (including heuristics of disability) are addressed within this stage by providing scope to discuss sense of identity, how this may be changing and what is important for the individual.

### Stimulating growth

Progressing along the EIVR continuum, EIVR providers continue to work with the individual to provide information and support to enable maintenance of self-confidence and self-efficacy with increasingly targeted conversations about options and possible RTW pathways. This enables the individual to reinforce their worker identity and map out possibilities for RTW, with the EIVR provider giving appropriate levels of support for each individual. While options can be explored within this stage, clarity about the individual’s function is important before pathways towards RTW can be developed or enacted.

### Communication: growth conditions

Communication with vocational consultants providing ongoing vocational support following discharge from EIVR is an important and necessary step. For those individuals who had progressed through the EIVR process, and developed possible pathways and options for RTW, it is important that timely and effective communication to a community-based vocational consultants occurs. This facilitates a seamless transition into further vocational rehabilitation.

### Communication: maintenance conditions

A strength of the EIVR programme is acknowledging an individual’s need to remain in any stage of the EIVR process for as long as required. This often means that an individual may not have transitioned through all the stages of the EIVR programme at the time of discharge from inpatient rehabilitation services. Thus, it is important that when the individual is discharged from inpatient rehabilitation and EIVR, timely communication to community vocational consultants occurs about where the individual is currently placed with regard to their RTW pathway. Information should also be provided on what the individual requires to ensure any progress during the EIVR programme is maintained and able to be continued with the new provider. We have articulated this in the graphic by the yellow arrows.

From this conceptual framework we articulated key processes and mechanisms (separated into resources and responses) that make up the elements of an EIVR programme (Table 3).

**Table 3:** Summary of key concepts and mechanisms for Early Intervention Vocational Rehabilitation

Key concepts	Mechanism	
	Resource	Response
Hope	Early conversations messaging that RTW possible.	Maintaining generalised sense of hope that work is possible at some point.
Preparing the team ground	RTW goals articulated as part of inpatient rehabilitation goals and reinforced in rehabilitation planning practices.	Consistent positive messaging from all rehabilitation team members about vocational plans and actions directed towards RTW across rehabilitation journey establishing and maintaining hope.
Priming the individual	Intentional but informal discussions with the individual identifying inherent individual potential, resources and strengths.	Engagement in RTW conversations (speed and direction governed by individual) by enabling the individual to determine priority of RTW-focussed goals within overall rehabilitation process.
Stimulating growth	Exploring options about work and mapping out possible pathways to enact vocational identity.	Developing pathways for RTW that are best aligned to individual’s capabilities, strengths and vocational identity.
Optimising individual conditions for growth	Identifying support or resources that need to be in place for individual to develop their own RTW-focussed goals and actions Exploration of internal schema of disability and psychological coping factors.	Improving self-efficacy and providing appropriate level of support to enable individual to enact pathways towards RTW goals. Changing sense of identity.
Communicating optimal conditions for current maintenance/future growth AND Communicating sustainable growth conditions	Ongoing vocational support on discharge from EIVR service to ensure growth is maintained and provide support when individual ready to establish more concrete RTW goals.	Provision of vocational service to continue to foster hope and keep individual thinking about RTW when they are discharged from EIVR.

Key: EIVR = Early Intervention Vocational Rehabilitation; RTW = return to work; VR = vocational rehabilitation

## Discussion

By synthesising data generated from literature review, survey, interviews and focus groups we have presented a refined conceptual framework describing the process of EIVR (Fig. 2) and have articulated the key mechanisms in the format of resources and responses that are required for EIVR to be successful. We have also used the refined programme theory to develop a figure based on a gardening metaphor, encapsulating the varying types and degrees of growth and support an individual needed in order to think about, plan and action RTW following SCI. The most important mechanism of any EIVR programme is the establishment and maintenance of hope by consistent messaging and continuity of messaging across the multi-disciplinary rehabilitation team that work is possible at some point following injury. Within EIVR, early provision of information, linking to resources and discussions of possibilities around RTW have been found to reinforce hope following SCI.[6] Further, linking this to Snyder’s Hope theory, establishing meaningful vocational goals and discussing possible pathways toward these goals would theoretically increase an individual’s hopefulness about RTW during rehabilitation.[21].

Other mechanisms include establishing a trusting relationship and getting to know the individual in order to provide an individualised and flexible approach to exploring biographical continuity, expectations of work within the wider cultural context, priorities, understanding of current capabilities and any internalised stigma around disability. EIVR providers gather information through informal but intentional conversations at a speed and direction governed by the individual. These conversations, along with conversations with the wider multidisciplinary team, may reduce biographical disruption and facilitate continuity of identity following SCI. Bourke et al. [22] postulated these can be restored to some degree by access to information, regaining control and restoring a sense of personal narrative, including where work fits into this narrative.

Worker identity can also be enhanced by maintaining connection with employers. Within the EIVR programme, EIVR providers can support the individual to maintain some connection with their employer. This also provides a conduit for generalised information exchange to improve the employer’s understanding of the individual’s injury and possible future sequalae. Bloom et al. [23] suggested that intrinsic motivation and personal beliefs about the value of working are components of occupational bond and this construct presents useful targets for intervention.

Further along the EIVR process, once there is clarity about the individual’s function, the mechanism of exploring options can be introduced to support the individual to explore, continually refine and eventually develop possible pathways towards RTW. The speed and direction of this needs to be led by the individual and impetus or momentum only provided when required. As the individual demonstrates an attitudinal shift away from optimism toward realism, then the EIVR provider can assist the individual in identifying obstacles to RTW and develop strategies or plans to mitigate these. This mechanism aims to empower the individual by targeting motivation and self-efficacy, but providing assistance if required.

The final mechanism of the EIVR programme in our conceptual framework, is communication with community based vocational providers. As the speed and direction of EIVR is led by the individual, each individual will be at a different point with regards to RTW at the time of discharge from the rehabilitation programme. Therefore, communication with community providers, irrespective of where the individual is in the EIVR process, is important to ensure ongoing momentum regarding RTW, through maintenance of hope and ongoing adjustment following injury.

Findings from this study have also been articulated in previous studies in the SCI population. In a qualitative study looking at barriers and facilitators to employment for people with SCI, Dorstyn et al. [24] recommended the following improvements in RTW services provided to people with SCI; explore why people want a job not just what job; individualised and personal approach, build rapport, presence of vocational rehabilitation staff on the rehabilitation ward, education of inpatient staff on importance of vocation as part of discharge planning; integration of vocational rehabilitation within medical rehabilitation, education about pathways to RTW, and advocacy about SCI needs to employers. These are all captured by our conceptual framework. The need for advocacy and skilled negotiation with employers when supporting a person with a neurological condition returning to work was also recommended by Harvey et al.[25] Findings from our survey study indicated that those with more autonomous roles such as senior management or professional roles had greater odds of returning to work sooner than those in less autonomous roles. This is consistent with previous research showing that having more education and a professional role prior to SCI can fast-track RTW [26] and reinforces the need to maintain relationships with employers. In a synthesis of the extant literature on RTW following SCI, Bloom et al. [27] found that self-efficacy, hope and motivation assisted in promoting psychological coping and well-being following injury, suggesting that these are useful factors to target in vocational rehabilitation.

As many people with SCI make decisions about employment within one month following injury [8], it is important that EIVR services be available as early as possible within inpatient rehabilitation services. However, the timing, speed and direction of the EIVR service is crucially important to the outcomes of the programme. Bloom [28] noted that readiness to think about work is not dichotomous, but rather a stage. Thus reinforcing the importance of the early mechanisms of EIVR, namely providing hope and gaining knowledge about the individual through informal but intentional conversations. This allows the vocational consultant the ability to determine what stage the individual is in with regards to thoughts about RTW and providing individualised and targeted intervention commensurate to the individual.

While not a limitation, it is important to note that the majority of participants in this study were covered by a no-fault cover compensation scheme managed by the government funded Accident Compensation Corporation. This scheme currently covers all medical expenses resulting from injury and compensates an individual up to 80% of their pre-accident earnings for as long as they are unable to return to paid employment. Under this scheme, people remain in rehabilitation until their goals (set with the rehabilitation team) are met rather than for a pre-defined time determined by the insurance provider. This means that following SCI the individual does not have any time pressures to RTW following injury. This may not be the case in other countries where time or financial pressures may be present. Having sufficient time to fully explore RTW options while receiving earnings compensation has been postulated to enhance RTW following injury.[29] One of the main limitations of this study is that the views of the employers of people with SCI who had received EIVR were not included. Future directions in research should include employers who have had input from EIVR providers to determine the value/benefit of such input.

## Conclusions

The use of realist research methods has allowed us to articular a more nuanced understanding of how EIVR work, for whom it works best, and the contexts promoting activation of RTW outcomes. While the mechanisms of EIVR have been postulated in previous literature, use of realist methods expands this by facilitating the explanation of the complex nature of RTW following SCI, including variation in outcomes in response to a range of programme resources. The mechanisms we have conceptualised within EIVR programme theory include the establishment and maintenance of hope, establishment of a trusting relationship that identifies an individual’s inherent potential, resources and strengths and providing appropriate support and resources for the individual as they progress through rehabilitation. Importantly, the speed, direction and nature of input provided by EIVR providers is governed by the individual and progression is reliant on clarity of function. By using a conceptual framework to describe EIVR programme theory this provides the opportunity to facilitate translation of this intervention into other populations facing similar impairments and barriers to RTW, such as stroke and traumatic brain injury populations.

## Electronic supplementary material

Below is the link to the electronic supplementary material.

Supplementary Material 1
