# Supplementary material for: Developing A Conceptual Framework for Early Intervention Vocational Rehabilitation for People Following Spinal Cord Injury
Source: J Occup Rehabil. 2022 Aug 4;33(1):179–88. doi: 10.1007/s10926-022-10060-9 (PMC10025215; doi:10.1007/s10926-022-10060-9)
Supplement: Supplementary file 1 — Supplementary Material 1 [file 10926_2022_10060_MOESM1_ESM.docx]

**Supplementary online 1: EVOCS Synthesis Matrix**

**Hope**

| **Context-Mechanism-Outcomes configurations** | **Key concepts** | **Supporting evidence** |
| --- | --- | --- |
| Constructing hope | The potential is within the individual but requires energy to commence and sustain change.  Needs to be present and fostered throughout rehab process. Important to consider role of hope around and after setbacks.  Considered necessary but not sufficient for all steps in the process  Can function as a context, a mechanism, and an outcome throughout the process.  EIVR –Identifying what may be needed between stages, prior to next vocational provider taking over and carrying on. | "the driving philosophy of EIVR was to instil the individual as early as possible, with a sense of hope and possibility about returning to or gaining work following SCI” (Johnston et al., 2016)  “The vocational coordinators seem to be directly and indirectly addressing the often unasked question of ‘will I be able to work again, by challenging and reframing ‘ability’. Hope was variously characterised as providing options, support, help, information, resources, pathways and possibilities, as well as creating expectations specifically with regards to vocation. ‘If we can early on plant that seed of hope and expectation that, you know, work is possible and often people are very positive in those early days. They have a lot of confidence about returning to work.” (Ramakrishnan et al., 2018)  “Themes that emerged from the data regarding the value of the service include hope (early after injury), a sense of direction with options” (Ramakrishnan et al., 2018)  “One of the most salient themes that emerged about early vocational rehabilitation was ‘hope’. The need, place, position and importance of providing hope at this early stage for those with SCI, were illustrated with metaphors such as ‘seed’, ‘flame’ and ‘door’. The vocational coordinators seem to be directly and indirectly addressing the often unasked question of ‘will I be able to work again, by challenging and reframing ‘ability’. Hope was variously characterised as providing options, support, help, information, resources, pathways and possibilities, as well as creating expectations specifically with regards to vocation. ‘If we can early on plant that seed of hope and expectation that, you know, work is possible and often people are very positive in those early days. They have a lot of confidence about returning to work. I think the risk is that the flame goes out.’ (VC2) ‘We basically introduce what we are, and I suppose, offer hope at an early stage at a time when a lot of the focus is on what someone has lost and it is at a time when they might be thinking “I will never be able to do this again. I will never be able to do that again.” (Hay-Smith et al., 2013)  *“To me, it’s as simple as, and it’s there, it’s confidence that I can return to work, but there’s also, but there’s also confidence that they will return to work, and they’re two different things.”* (EIVR FG participant #5).  *“ …. because it, at that point I thought everything was pretty much over and to have [the EIVS provider] come in and show me that there is possibilities of, of work, and possibilities of things I can do, was good. It was good.” [SCI28]*  64.9% of survey respondents indicated that they “felt hopeful about returning to work while I was in the spinal unit”. Similarly, 64.9% stated that they “expected to return to work after my SCI” |

**Preparing the team ground**

| **Context-Mechanism-Outcomes configurations** | **Key concepts** | **Supporting evidence** |
| --- | --- | --- |
| Shifting rehab team culture  Employment discussed as an expected outcome | Rehab environment allows for RTW conversations from early in the process. RTW goals and consideration of this process set from the beginning.  Positive expectations of team.  Wider contextual considerations also included, e.g., policy & funding considerations  Rehab team culture and any cultural change/development – including acceptance of the vocational provider and the opportunity of them to do their role. | The inclusion and integration of VR into inpatient settings seemingly introduced an element of hope and support that encouraged patients to see the possibility of returning to some form of work and/or study in the future, with observable improvement in patient engagement in other aspects of inpatient rehabilitation” (Ramakrishnan et al., 2016)  “Another insight from the research findings is that these influences on decisions about employment seem to become relevant fairly soon after injury, with some influences, particularly those related to perceptions, probably beginning to develop from quite early on (for example: self confidence, perceptions about physical capability, advocacy and support available). This suggests a need for vocationally focused support services to be made available as early as practicable, even integrated with other rehabilitation services.”(Fadyl & McPherson, 2010)  “Although early intervention and integration of services within the primary rehabilitation programme appears to have many positive effects, the willingness of participants to engage in such a programme soon after their injury is variable. Qualitative research reveals varying levels of motivation, with some participants unwilling to approach topic of returning to work during a time of tremendous physical, psychological and interpersonal upheaval. This is congruent with the predominant thinking surrounding returning to work post SCI and has important implications for any model attempting to incorporate an early intervention approach, which must consider appropriate timing and assertiveness of intervention” (Bloom et al., 2017)  “The attitudes and motivations of treating clinicians are just as vital as those of the clients to the success of early vocational intervention. Many clinicians may subscribe to traditional thought regarding SCI rehabilitation and may reject vocational intervention at such an early stage. One strategy to overcome this is integration of the VR professional within the primary rehabilitation team. This may foster cultural change within the team and significantly enhance client access to VR services.” (Bloom et al., 2017)  *“I think the existence of the services in both places, and people that have chatted to me that it’s, you know, more modern or whatever, I don’t believe that perception exists anywhere near in the same way as it did. I don’t, you know, and even then, it was probably fifty-fifty. Now I don’t believe it really exists, so I think the, the buy-in for the staff is just, just a given. There’s just no doubt that, that if any clinical staff think that the conversation about work or setting goals based on work, would be helpful, they’d embrace it, they’d totally support that, no matter who the client was or what state or how severely disabled they may be perceived. I think, I think, I don’t get a sense of any clinical staff with any pushback on that.”* (EIVR FG participant #2). |

**Priming the individual seed**

| **Context-Mechanism-Outcomes configurations** | **Key concepts** | **Supporting evidence** |
| --- | --- | --- |
| Psychological adjustment  Transitioning/Importance of the approach  Instagram travel inspiration – establishing a therapeutic relationship | Someone I trust & potential value of lived experience  Creating optimal conditions for individuals to be able to work towards addressing RTW goals.  Motivational interviewing and stages of change  Relationship development between vocational consultant and individual  Dedicated role for vocational rehabilitation  Different levels of preparation for different individuals, but an environment to support differences in readiness and adjustment.  Accepting the individual can stay in the space for as long as needed. | “Importance was placed on sensitively introducing patients to InVoc. Assessing a patient’s readiness to participate in the InVoc programme was a considered process, determined on a case-by case basis, and involved consultation with all staff in case conferences, as well as informal conversations with a patient’s family” (Johnston et al., 2016)  *“They might say it. ‘No, I wanna focus on my, on my physical rehab at the moment.’ They actually say that a lot. ‘I’m going to the gym. That’s what I’m thinking about, focusing on my walking. I’m not thinking about work yet. No, that’s down the track. Yes, I will, but down the track.’ They’ll say things like that.”* (EIVR FG participant #2).  *“There’s got to be a good relationship there. Lots of good conversations that aren’t even just voc [sic], other ones.*  *FG participant 1: We have lots of conversations that aren’t actually voc [sic], but then it kind of is because some stuff needs to have a rapport.*  *FG participant 2: Before voc [sic], yeah. The same with education, you know like education, like no one’s gonna go back to work, if they can’t work out how to, you know, do these other things, and they’re not all just physical rehab kind of things.”* (EIVR FG participant 1 &2).  *“The advantage of the early intervention is setting hope. That’s go away and come back to another provider. Coming back to us is great, because we’ve already got a relationship, and that’s the ideal, and you guys are really lucky with that ‘cos building on that relationship must be very useful for the person. They don’t have to go over their story again, you kind of already know them, their story, their family, their history, what they’ve been through, being here while they’ve gone, you know, done their rehab. The relationship is in the trust they have in you, I think, is important, and it can be quite, you know, in certain cases especially for us, when we don’t get them back, and they go off to someone else.”* (EIVR FG participant #2).  *“ Yeah, so you know, so, ‘cos I think, you know, if I remember my first conversation with [Vocational Consultant] was yeah, it was that negative, it wasn’t funny, you know? But as we’ve got, got on, it’s getting more positive, more positive…. I think she was more listening than anything, you know. I think it was, when we first started conversation, it was that we were more, she was more the listener than the thing, so it was the second sort of conversation we had that we started putting these little drives in place, you know what I mean? I had more of a positive thought on life by then.”[SCI22]*  *“ She’d almost hunt me down every day and have a talk. But a lot, I wasn’t really focusing on work then, ‘cos I knew like I was there to work on myself. I wasn’t really focusing on work, but me and her, she wrote a CV up for me and all that sort of stuff. So there was a bit of help when I was in rehab. I honestly wasn’t too interested in work at that point.” [SCI31]*  Importance of timing … but creating opportunities to start the conversations  *“ Too early. I think it was a bit too early, yeah, ‘cos my, I sort of had to think and stop and think about all the things. There was a lot of things going through my mind at the time….All I wanted to do was really hurry up and get out of the unit. “ [SCI29]*  Discrepancy in individual view about timing *I believe it is too early to have a chat regarding return to work at the spinal unit. Your whole life has just changed. The best time would be to approach the individual once they are settled at home, therefore more comfortable to talk about future options*. (Survey ID # 26, female, 50y) – in contrast 51.4% stated that they had been approached about this at the right time.  37.8% of survey sample agreed that “NZST helped me to think about returning to work by sharing others’ stories |

**Optimising unique conditions for growth**

| **Context-Mechanism-Outcomes configurations** | **Key concepts** | **Supporting evidence** |
| --- | --- | --- |
| Building self-efficacy  Booking Online Travel Agent  (resource: identification of supports available and what is required)  Building self-efficacy  Psychological adjustment  Maintaining worker identity  (resource: to improve self-efficacy) | Exploration of self, work, priorities, abilities, resilience and strengths. Scope to discuss sense of identity and how this may be changing, what is important  What supports are needed to promote self-efficacy and retention of identity in worker role. Different amounts of support or scaffolding required for different individuals. Natural climbers (high self-efficacy) will utilise minimal input compared to those who require extensive support and lack autonomy in action (low self-efficacy).  Giving individuals the support to manage any setbacks  Space to receive support following setbacks when too much has been attempted (flourishing)  Individualised and strengths based approach to the individual.  Internal schema and psychological coping factors (including heuristics of disability)  Contexts including who the individual is; who they want to be; lived experiences.  Importance of clarity about functioning > can’t move to differentiation/establishment phase without this happening | “Moderators of success predominantly pertained to physical impairments and whether these were perceived as a work barrier, and psychological factors including self-concept, role modelling, optimism, coping and motivation.” (Hay-Smith et al., 2013)  “During rehabilitation the therapist acted as an external motivator to the participants to self-reflect on their feelings, identify strengths and establish an awareness of their limitations, which in turn enabled them to formulate more realistic goals for themselves, which is part of establishing a positive self-efficacy.” (Soeker & Darries, 2019)  “The vocational coordinators seem to be directly and indirectly addressing the often unasked question of ‘will I be able to work again, by challenging and reframing ‘ability’. Hope was variously characterised as providing options, support, help, information, resources, pathways and possibilities, as well as creating expectations specifically with regards to vocation. ‘If we can early on plant that seed of hope and expectation that, you know, work is possible and often people are very positive in those early days. They have a lot of confidence about returning to work.” (Ramakrishnan et al., 2018)  “…people who are stricken by a catastrophic change go through an adjustment process in which they have to handle the feeling of loss and interruption from their earlier life, as well as the anger, frustration, fear, and/or depression it causes. Not until they have done so are they able to look forward and take the first step in their adjustment process. After a severe injury implying great changes to the individual, it is important to obtain help to cope with the sadness of having lost abilities, and to gain insight into how the injury affects one’s everyday life.” (Rubenson et al., 2007)  *“She’s extremely helpful, friendly lady who’s easy to talk to and is very helpful. At a time where I am ready to take my next step, as far as work goes, [Vocational Consultant] will be there.” [SCI28]*  *“ I’ve managed that relationship [with my employer] myself and if it comes time that I think I can do something at work, I’ll broach that subject. If not, and if something comes up elsewhere, I’ll discuss that with [Vocational consultant].” [SCI28]*  High Self-efficacy:  *I mean, and I don’t wanna sort of down cry the role or anything, but yeah, for me, personally, it wasn’t a sort of an area that I needed a lot of help and support with. [SCI12]*  Clarity (but also prioritisation in constellation of concerns)  *“Ah, I suppose, like the transferring stuff, if I was able to nail those sort of physical tasks, you know, it would make me feel a lot better. Because that means I could get up and go whenever I felt like it.”[SCI04]*  Participants described the need to rebuild confidence about their capabilities in order to consider RTW but that this can take time and is a slow process:  *It's really hard; best to go as slowly as possible and be as open about what you can and can't do [NZST staff] from [spinal unit] still help me with work questions and helped me so much to get confident that getting back into work was viable*. (Survey ID # 7, female, 40y).  *It is a lot harder than can be imagined, (RTW) requires confidence and need to start slow on low hours first*. (Survey ID # 24, male, 66y)  Responses to the closed survey questions echoed these sentiments. For example, many participants felt EIVR input helped them feel confident and optimistic about returning to work (59%) and assisted them work through hurdles and options (59%). |

**Stimulating Growth**

| **Context-Mechanism-Outcomes configurations** | **Key concepts** | **Supporting evidence** |
| --- | --- | --- |
| Exploring options  Maintaining worker identity  Mapping out possible itineraries | Exploring what options are available and looking for a space to flourish, mapping out possibilities with differing levels of support as required.  Self-confidence and confidence in abilities maintained | “Not only a sense of direction, but also distraction and having choices seemed important for participants to feel empowered, and were reflected in comments such as “… gave me options…good options…other directions to look into … … the more options you’ve got while you’re in places like this, the better …it was good to keep me mind on something other than what was going on...… the really helpful things were providing me with details of, you know, the various support services at uni and – and that, you know, studying was a possibility”(Ramakrishnan et al., 2016)  “The In-Voc model seemed to have empowered and motivated individuals, building confidence that RTW is a realistic rehabilitation outcome, by focussing on achieving relevant goals, participating in successful experiences while in hospital, providing support to make informed decisions and developing an awareness of adaptations, modifications and assistive devices.” (Middleton et al., 2015)  *“… just informing me of the options I do have out there, if I needed to. She understood I’d just arrived there, but she gave me things to think about for the future, for if I did well in my recovery and stuff and I was thinking about looking for work, what kind of work I would like to do, and how to achieve that…. she told stories and she showed pamphlets that people have, stories that they have shared, so those sort of things were helpful.” [SCI04]*  Maintaining and developing confidence  *“…. I guess doing what she’s doing now, giving people confidence about doing things. You know, because each person’s different, I guess, you know, have different kind of drives, yeah, no, I think she’s got an important job….. she built my confidence up….”[SCI04]*  *“…. like I’m quite lucky, I’ve got options and all that sort of stuff, but people with, maybe with not so many options, it would be really handy for people that have, for someone like [EIVR provider] to come up to them and say, ‘Hey, I’ve got someone like you into a job like this,’ sort of thing, you know, help them out…. people see so many reasons why they can’t go back to work, and it’s good to hear that people, you know, that they can. It’s good. Especially younger people that maybe haven’t worked before, or just kind of started working…..” [SCI31]*  *“….I’ve got a pretty good focus on what I want to do. You know what I mean, I’m not, I just take each day as it arrives, I don’t look too far ahead of myself. But yeah, so at the moment, the thought at the moment is, I’ll just take each day as it arrives and see how it goes. If I’m feeling in the, I want to go fencing, I’ll go fencing, yeah, and yeah, but other than that, I’ll just cruise it. ….I can’t see me going back into the cowshed before Christmas, but after Christmas, I could possibly see me going back into there, once my strength’s built up, and my idea is that I go, it’s gonna cost the farm, I think, because I’ll be on ACC, so I’ll just go there and just do their fencing, just build my strength up again, you know.” [SCI22]* |

**Communicating growth conditions**

| **Context-Mechanism-Outcomes configurations** | **Key concepts** | **Supporting evidence** |
| --- | --- | --- |
| Booking online  Travel agent  Staying Connected  Maintaining worker identity  Changing perceptions  (resource: communication between patient and employer) | Path out of rehab environment into real world  Employer’s expectations and staying connected with employer and colleagues  Facilitates movement between different stages of rehabilitation.  Collaboration between involved parties  Relationships and interactions with rehabilitation team and employer | “Also, as advocated by the Vocational Case Coordination model, providing early links to employers that provide support and education may help with availability of suitable work, and keep the individual themselves thinking about a RTW as possible and beneficial.” (Fadyl & McPherson, 2010)  “Contact with the previous employer would involve providing information regarding the injury and the likely resulting physical impairment, information regarding available workplace supports or incentives available to the employer, and a discussion about the availability of suitable work tasks for the employee. This highlights an opportunity for the involvement of vocational specialists, such as rehabilitation counsellors, whose niche skill set is typically underutilised within the primary rehabilitation context and whose professional management of this sensitive task could preserve jobs that would otherwise be lost.” (Bloom et al., 2017)  “Integrating vocational rehabilitation within the inpatient context could also preserve the occupational bond by reinforcing the plausibility and primacy of work after SCI” (Bloom et al., 2019)  “*If they do want to go back to work, then talking to that employer and educating that employer can save that job for later, because that employer is only hearing ‘I don’t know how long I’ll be in hospital. I don’t know if I’ll walk again. I don’t know if I’m coming back. I don’t …’ There’s all these, ‘I don’t knows’, the employer has no idea, and sometimes we can be that middleman and just say, ‘Hey, they’re likely to be here for four months and they’re likely to need to come back part-time and work their hours up, and this is what it looks like, and this is the support that’s around these things, so it’s really early days and we don’t know (?)’ So, then usually the employer like really, you know, appreciates that, because then they know, for their business, whether they need to get a casual worker in, a part-time worker, you know, what they’re sort of getting cover for, because sometimes they really don’t know*” (EIVR FG participant #2).  A small number of survey participants endorsed assistance to engage with the employer as helpful (19%). No participants agreed that EIVR input involved liaising with the employer on their behalf, consistent with the stated intentions of the NZST vocational service as supporting their clients to do as much of the work as possible.  Confusion about the RTW process was also identified. For example, in relation to conflicting expectations between the individual and various organisations involved: *As I was an employee of [large organisation] my return to work was handled by [in house team]. I felt this excluded input from Spinal Trust and different expectations were confusing.* (Survey ID # 38, female, 56y) |

**Communicating maintenance conditions**

| **Context-Mechanism-Outcomes configurations** | **Key concepts** | **Supporting evidence** |
| --- | --- | --- |
| Flexing work roles  Building self-efficacy  Engaging in solution-focused work options  Checking for travel alerts  (resource: discussion that supports these outcomes of flexing work roles and solution focused work options – mechanism is building self-efficacy) | RTW support within context of known work role.  Social learning theory and modelling how to manage post-injury  Flexing work roles and practical adaptations to position – planning rather than enacting.  Consideration of modifications to work environment or conditions to enable RTW to be initiated.  Checking realistic expectations  Look for solution focused work options that will work for them – considering flexing work roles  Resources are discussions supporting these outcomes | “This is primarily through empathy for the injured worker and acceptance of the adjustments made to accommodate them. Job accommodations and stigma about injured workers can contribute to unsupportive or antagonistic co-worker relationships, which diminish employment outcomes” (Bloom et al., 2019)  Contact with EIVR once home  *“…. Chatting through what it might be, and [EVIR provider] still contacts me. She still contacts me to chat, chat about things and sees how I am, sees how I am and whatnot. Like our relationship hasn’t stopped at the spinal unit. It’s carried on to today, you know.” [SCI28]*  This was largely unsupported by the survey responses, NZST helped me talk to my employer (0%); NZST talked to my employer on my behalf (18.9%); - NZST helped me to understand administration processes for return to work (32.4%).  However, higher rates of agreement were seen for - NZST helped me work through future return to work options (59.5%) |

**Text colour key:** Black = from literature review; Blue = from interviews of people with SCI; Green = from survey of people with SCI; Purple = from focus groups

**Abbreviations** EIVR = early intervention vocational rehabilitation; FG = focus group; NZST = New Zealand Spinal Trust; RTW = return to work; SCI = spinal cord injured participant.

**References:**

Bloom, J., Dorsett, P., & McLennan, V. (2017). Integrated services and early intervention in the vocational rehabilitation of people with spinal cord injuries. *Spinal cord series and cases, 3*(1), 1-4.

Bloom, J., McLennan, V., & Dorsett, P. (2019). Occupational bonding after spinal cord injury: A review and narrative synthesis. *Journal of Vocational Rehabilitation, 50*(1), 109-120. <https://ovidsp.ovid.com/ovidweb.cgi?T=JS&CSC=Y&NEWS=N&PAGE=fulltext&D=emexa&AN=626247858>

Fadyl, J. K., & McPherson, K. M. (2010). Understanding decisions about work after spinal cord injury. *Journal of Occupational Rehabilitation, 20*(1), 69-80.

Hay-Smith, E. J., Dickson, B., Nunnerley, J., & Sinnott, K. A. (2013, Aug). "The final piece of the puzzle to fit in": an interpretative phenomenological analysis of the return to employment in New Zealand after spinal cord injury. *Disability and Rehabilitation, 35*(17), 1436-1446.

Johnston, D., Ramakrishnan, K., Garth, B., Murphy, G., Middleton, J. W., & Cameron, I. D. (2016, 12 Oct). Early access to vocational rehabilitation for inpatients with spinal cord injury: A qualitative study of staff perceptions. *Journal of Rehabilitation Medicine, 48*(9), 776-780.

Middleton, J. W., Johnston, D., Murphy, G., Ramakrishnan, K., Savage, N., Harper, R., Compton, J., & Cameron, I. D. (2015). Early access to vocational rehabilitation for spinal cord injury inpatients. *Journal of Rehabilitation Medicine, 47*(7), 626-631.

Ramakrishnan, K., Johnston, D., Garth, B., Murphy, G., Middleton, J., & Cameron, I. (2016). Early access to vocational rehabilitation for inpatients with spinal cord injury: A qualitative study of patients' perceptions. *Topics in Spinal Cord Injury Rehabilitation, 22*(3), 183-191.

Ramakrishnan, K., Murphy, G., Middleton, J., & Cameron, I. (2018). Early vocational rehabilitation for patients with spinal injury: A qualitative research study of service providers. *International Journal of Therapy and Rehabilitation, 25*(10), 505-515. <https://doi.org/http://dx.doi.org/10.12968/ijtr.2018.25.10.505>

Rubenson, C., Svensson, E., Linddahl, I., & Björklund, A. (2007). Experiences of returning to work after acquired brain injury. *Scandinavian Journal of Occupational Therapy, 14*(4), 205-214.

Soeker, M. S., & Darries, Z. (2019). The experiences of women with traumatic brain injury about the barriers and facilitators experienced after vocational rehabilitation in the Western Cape Metropole, South Africa. *Work (Reading, Mass.), 64*(3), 477-486.
